# Supplementary material for: Pharmacologically inducing regenerative cardiac cells by small molecule drugs
Source: eLife. 2024 Dec 9;13:RP93405. doi: 10.7554/eLife.93405 (PMC11627505; doi:10.7554/eLife.93405)
Supplement: Figure 1—source data 1. [file elife-93405-fig1-data1.zip › Figure 1-source data 1.pdf]

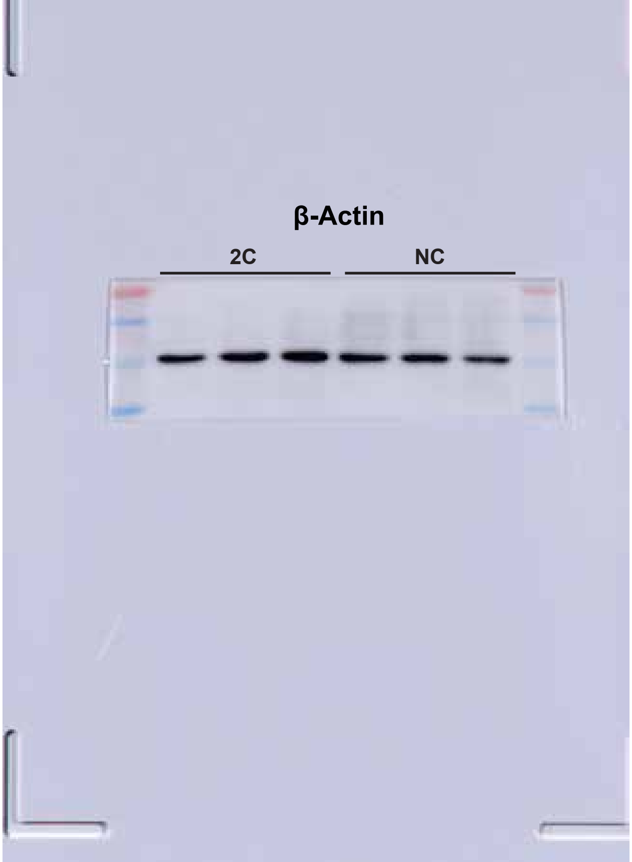

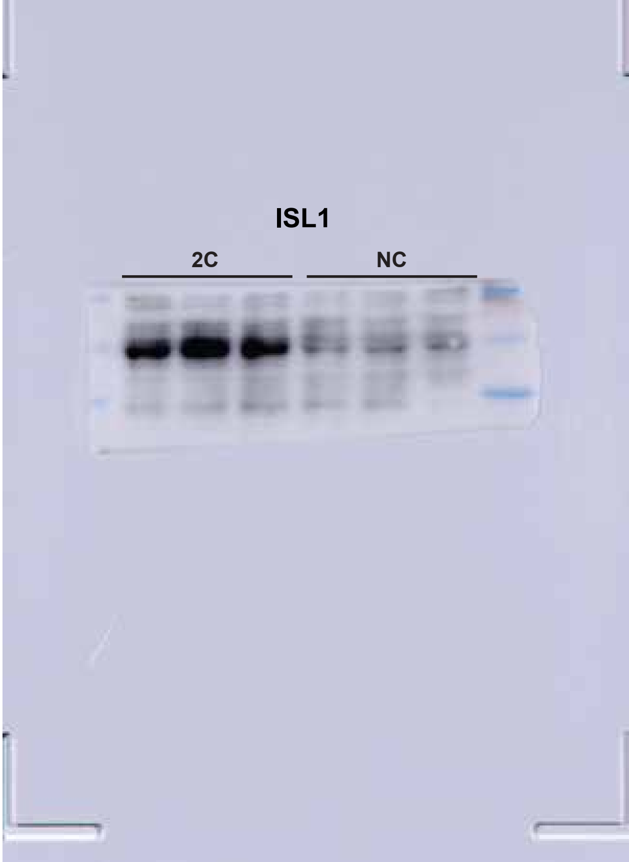

Figure 1, Source Data 1. Original western blot corresponding to Figure 1F. Western blot shows the expression of β-Actin (left panel) and ISL1 (right panel) in DMSO (NC) or 2C-treated CMs for 60 hours.
