# Supplementary figures and images for: Pharmacologically inducing regenerative cardiac cells by small molecule drugs
Source: eLife. 2024 Dec 9;13:RP93405. doi: 10.7554/eLife.93405 (PMC11627505; doi:10.7554/eLife.93405)

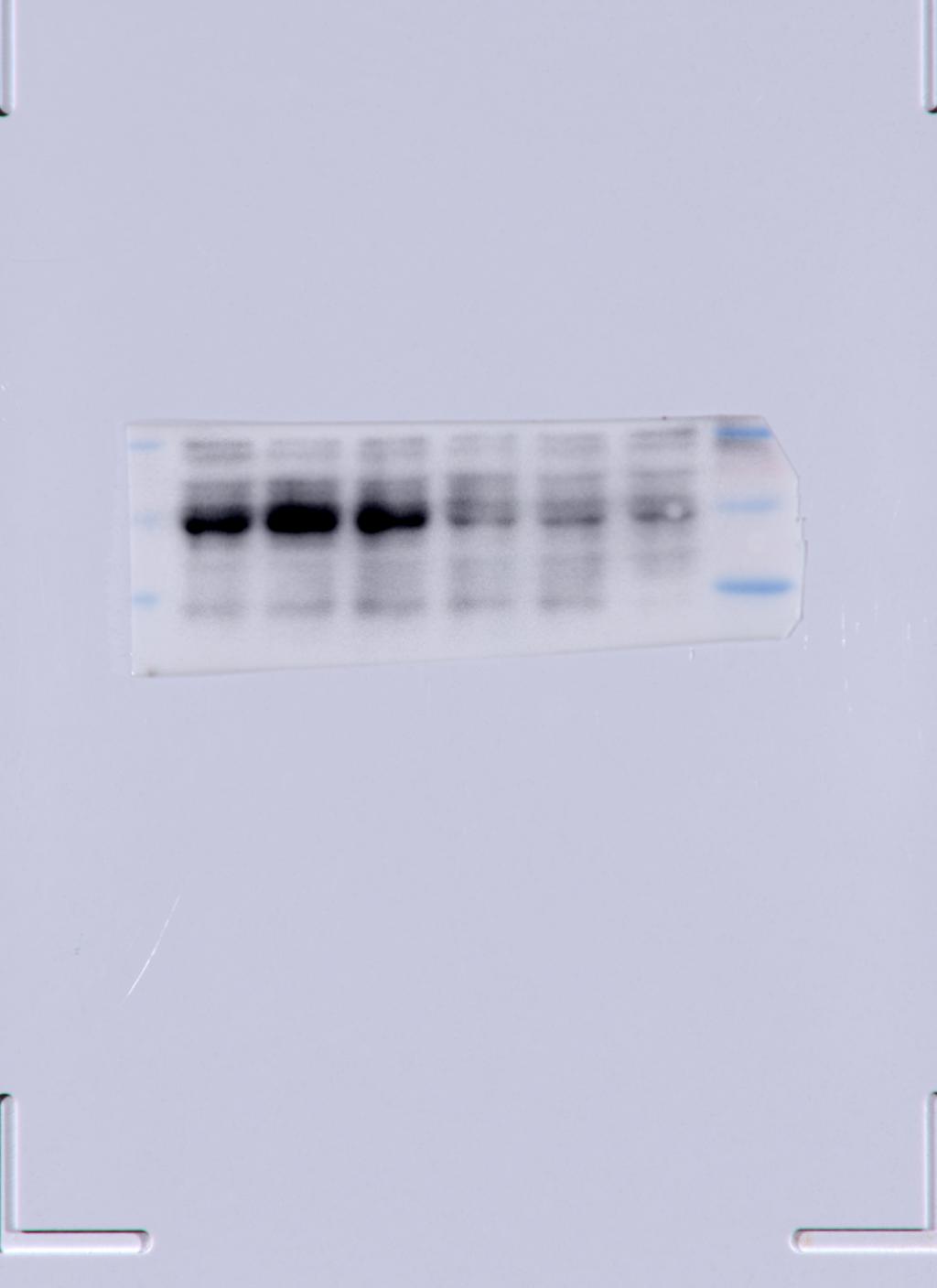

Supplement: Figure 1—source data 2. [file elife-93405-fig1-data2.zip › Figure 1-source data 2./ISL1.jpg]

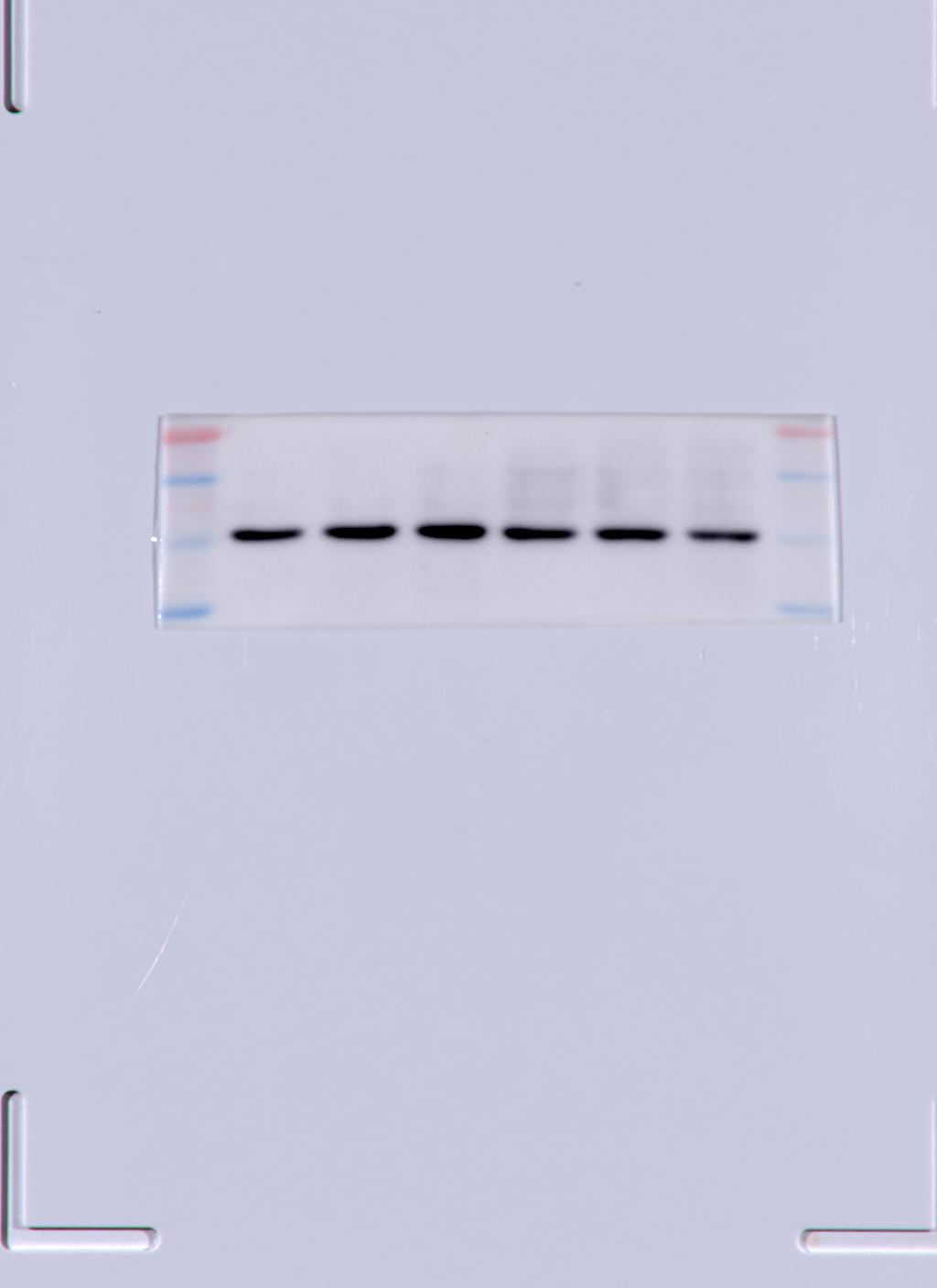

Supplement: Figure 1—source data 2. [file elife-93405-fig1-data2.zip › Figure 1-source data 2./╬▓-Actin.jpg]

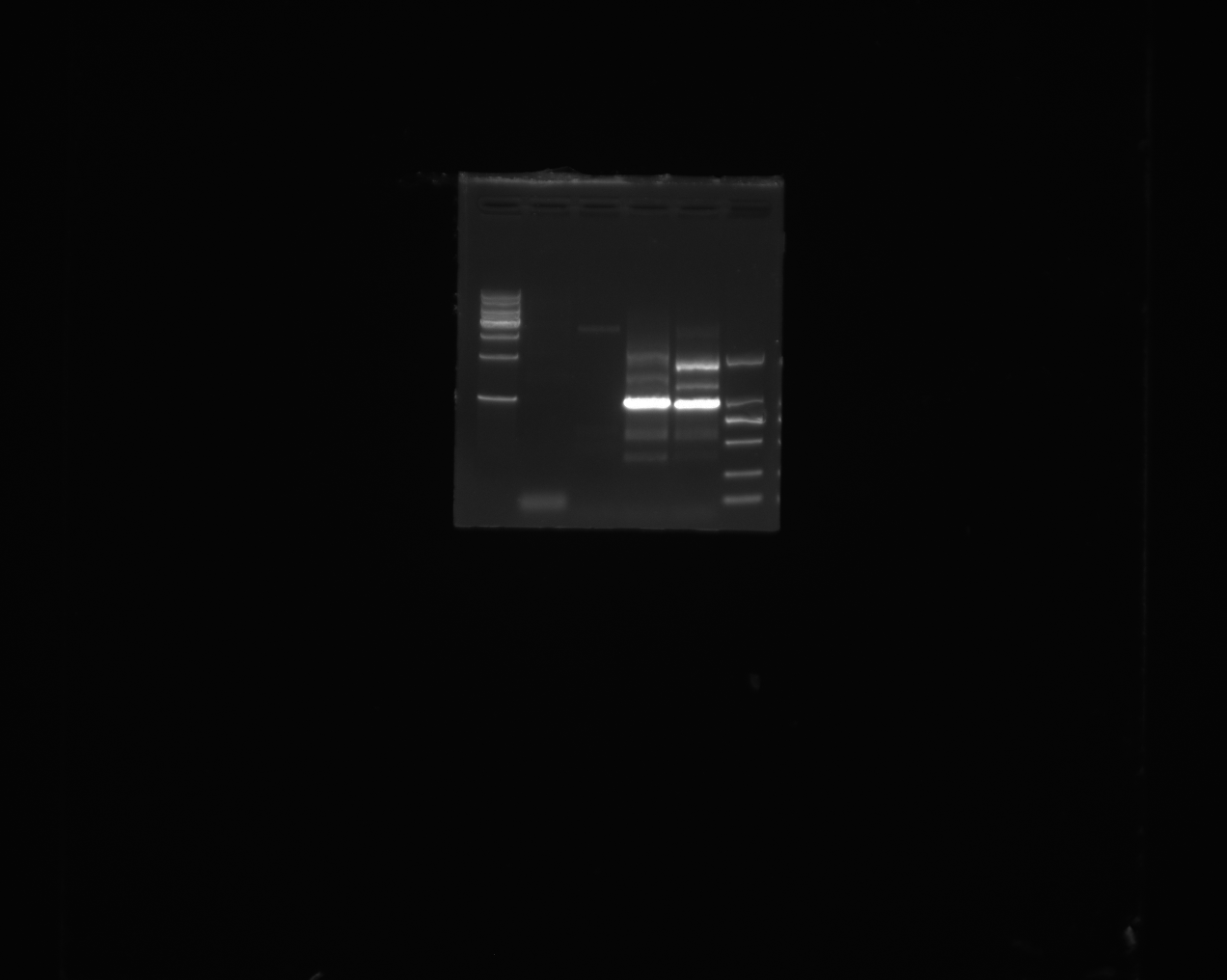

Supplement: Figure 3—figure supplement 1—source data 2. [file elife-93405-fig3-figsupp1-data2.zip › Figure 3ΓÇöfigure supplement 1-source data 2.tif]
