# Supplementary material for: Pharmacologically inducing regenerative cardiac cells by small molecule drugs
Source: eLife. 2024 Dec 9;13:RP93405. doi: 10.7554/eLife.93405 (PMC11627505; doi:10.7554/eLife.93405)
Supplement: Figure 3—figure supplement 1—source data 1. [file elife-93405-fig3-figsupp1-data1.zip › Figure 3ΓÇöfigure supplement 1-source data 1.pdf]

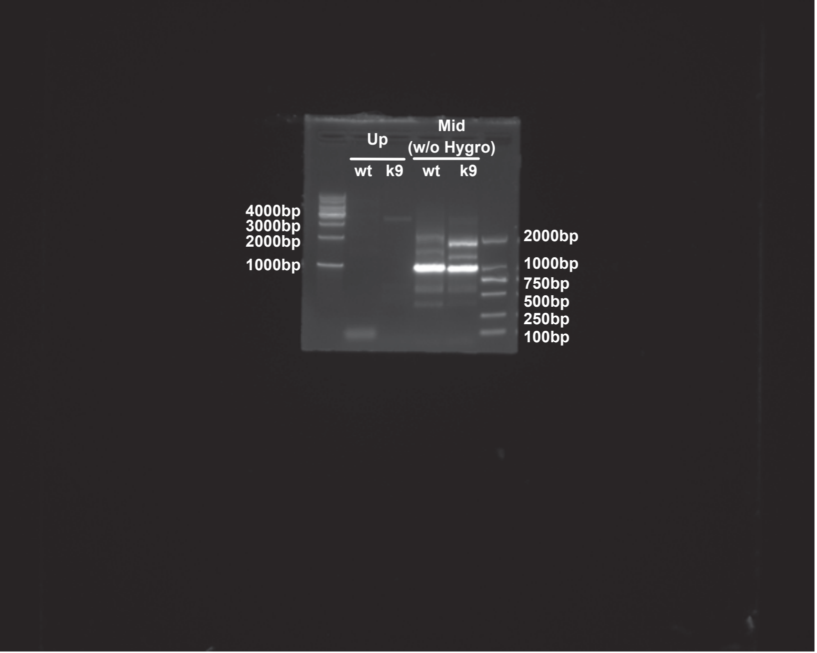

Figure 3, figure supplement 1, source data 2. Original gel image corresponding to Figure 3—figure supplement 1B. Genomic PCR confirmed establishment of heterozygous cell lines with ISL1-mCherry knocked in the targeted ISL1 locus.
