# Supplementary material for: Pharmacologically inducing regenerative cardiac cells by small molecule drugs
Source: eLife. 2024 Dec 9;13:RP93405. doi: 10.7554/eLife.93405 (PMC11627505; doi:10.7554/eLife.93405)
Supplement: Supplementary file 1. — The compounds from the lab’s proprietary library, along with their respective targets and working concentrations, are provided in the table. [file elife-93405-supp1.docx]

| **Tables 1. Compound library collected based on hypotheses.**   \| **No.** \| **Full Name** \| **Function (s)** \| **Concentration (µM)** \| \| --- \| --- \| --- \| --- \| \| 1 \| RG108 \| DNMTs inhibitor \| 0.04 \| \| 2 \| Decitabine \| DNMTs inhibitor \| 2 \| \| 3 \| RSC133 \| DNMTs inhibitor \| 10 \| \| 4 \| DZNeP \| DNMTs inhibitor \| 0.05 \| \| 5 \| Azacitidine (5AzaC) \| DNMTs inhibitor \| 2 \| \| 6 \| Trichostatin A (TSA) \| HDACs inhibitor \| 0.005 \| \| 7 \| Sodium butyrate (NaB) \| HDACs inhibitor \| 250 \| \| 8 \| Valproic acid sodium salt \| HDACs inhibitor \| 500 \| \| 9 \| Vorinostat (SAHA) \| HDACs inhibitor \| 5 \| \| 10 \| RGFP966 \| HDACs inhibitor \| 1 \| \| 11 \| Romidepsin (FK228) \| HDACs inhibitor \| 1 \| \| 12 \| C646 \| HAT inhitibor \| 2 \| \| 13 \| BIX01294 \| HMTs \| 2 \| \| 14 \| Tranylcypromine HCl (Parnate) \| HMTs \| 5 \| \| 15 \| EPZ004777 \| HMTs \| 5 \| \| 16 \| SGC 0946 \| HMTs \| 5 \| \| 17 \| GSK126 \| HMT inhibitor \| 10 \| \| 18 \| GSK-LSD1 2HCl \| HMT inhibitor \| 10 \| \| 19 \| ML324 \| HDM inhibitor \| 5 \| \| 20 \| GSK J4 HCl \| HDM inhibitor \| 1 \| \| 21 \| SB431542 \| TGF-b inhibitor \| 3 \| \| 22 \| RepSox \| TGF-b inhibitor \| 10 \| \| 23 \| A 83-01 \| TGF-b inhibitor \| 1 \| \| 24 \| LY-364947 \| TGF-b inhibitor \| 5 \| \| 25 \| LDN-193189 \| BMP signaling inhibitor \| 0.5 \| \| 26 \| PD0325901 \| MAPK/ERK inhibitors \| 1 \| \| 27 \| Forskolin \| PKA activators \| 10 \| \| 28 \| PS48 \| PI3K/Akt \| 5 \| \| 29 \| CHIR99021 \| Canonical Wnt \| 5 \| \| 30 \| Apigenin \| a potent P450 inhibitor for CYP2C9 \| 10 \| \| 31 \| BIO \| GSK-3 Inhibitor \| 0.1 \| \| 32 \| Y27632 2HCl \| ROCK inhibitors \| 10 \| \| 33 \| Thiazovivin \| ROCK inhibitor \| 0.5 \| \| 34 \| TTNPB \| Nuclear Receptor \| 1 \| \| 35 \| AM580 \| retinoic acid receptor agonist \| 0.01 \| \| 36 \| Nocodazole (NC) \| Cell-cycle inhibitors \| 0.1ug/ml \| \| 37 \| NU6140 (NU) \| ATR/CDK inhibitor \| 2 \| \| 38 \| Dasatinib \| Src Family Kinase inhibitors \| 0.5 \| \| 39 \| PP1 \| Src inhibitor \| 5 \| \| 40 \| Torin1 \| Potent and selective mTOR inhibitor \| 1 \| \| 41 \| (R)-(+)-Bay K 8644 \| L-type Ca2+-channel blocker \| 2 \| \| 42 \| Kenpaullone \| GSK3-beta and CDK inhibitor \| 5 \| \| 43 \| Compound E \| Notch signaling suppressor \| 0.1 \| \| 44 \| Vitamin C \| Others \| 25 ug/ml \| \| 45 \| L-Ascorbic acid 2-phosphate \| stimulate collagen formation \| 25 ug/ml \| \| 46 \| Tretinoin (RA) \| a ligand for both the retinoic acid receptor (RAR) and the retinoid X receptor (RXR) \| 0.25 \| \| 47 \| Hh-Ag1.5 \| Hedgehog Agonist \| 0.5 \| \| 48 \| Z-VAD-FMK \| Cell Death inhibitor \| 20 \| \| 49 \| 3-Methyladenine \| Cell Death inhibitor \| 5mM \| \| 50 \| Necrostatin-1 \| Cell Death inhibitor \| 30 \| \| 51 \| Liproxstatin-1 \| Cell Death inhibitor \| 0.2 \| \| 52 \| Y27632 \| Cell Death inhibitor \| 10 \| \| 53 \| Rapamycin \| Cell Death inhibitor \| 0.1 \| \| 54 \| IM-54 \| Cell Death inhibitor \| 10 \| \| 55 \| diphenyl-benzoquinone (DPQ) \| Cell Death inhibitor \| 1 \| \| 56 \| SU5402 \| receptor tyrosine kinase inhibitor（VEGFR2、FGFR1、PDGFRβ） \| 10 \| \| 57 \| L-NAME HCl \| NO synthase inhibitor \| 100 \| \| 58 \| JAK inhibitor I \| An ATP-competitive inhibitor of Janus protein tyrosine kinases (JAKs). \| 1 \| \| 59 \| SC1 (Pluripotin) \| Dual inhibition of ERK1 and Ras GTPase \| 1 \| \| 60 \| PD173074 \| FGF receptor inhibition \| 0.5 \| \| 61 \| SU16F \| PDGFR-β inhibition \| 2 \| \| 62 \| JNJ10198409 \| Dual inhibition of PDGFR-α and PDGFR-β \| 0.1 \| \| 63 \| DAPT \| Notch inhibition \| 1 \| \| 64 \| LY-411575 \| Notch inhibition \| 0.01 \| \| 65 \| Purmorphamine \| Hedgehog activation \| 1 \| \| 66 \| Prostaglandin E2 (PGE2) \| PKA activation \| 1 \| \| 67 \| IBMX \| PKA activation \| 10 \| \| 68 \| CD437 \| RAR activation \| 0.1 \| \| 69 \| Bexarotene \| RAR activation \| 5 \| \| 70 \| HX531 \| RAR activation \| 1 \| \| 71 \| 9-cis-RA \| Dual activation of RAR and RXR \| 2 \| \| 72 \| GW501516 \| PPARβ activation \| 0.1 \| \| 73 \| Carbacyclin \| PPARβ activation \| 10 \| \| 74 \| IKK 16 \| IKK inhibitor \| 0.2 \| \| 75 \| SC-514 \| IKK inhibitor \| 3 \| \| 76 \| PF184 \| IKK inhibitor \| 0.2 \| \| 77 \| Poly (I:C) \| Toll-like receptor 3 (TLR3) activation \| 300 ng/ml \| \| 78 \| Zebularine \| DNA methyltransferase inhibition \| 100 \| \| 79 \| UNC0638 \| G9a and GLP histone methyltransferase inhibition \| 0.5 \| \| 80 \| Chaetocin \| Histone methyltransferase inhibition \| 2 \| \| 81 \| PRT 4165 \| Polycomb repressive complex 1 inhibition \| 10 \| \| 82 \| IOX1 \| JMJC histone demethylase inhibition \| 1 \| \| 83 \| Tubastatin A \| Histone deacetylase inhibition \| 0.5 \| \| 84 \| MS-275 \| Histone deacetylase inhibition \| 1 \| \| 85 \| TC-H 106 \| Histone deacetylase inhibition \| 1 \| \| 86 \| MC1568 \| Histone deacetylase inhibition \| 2 \| \| 87 \| PCI 34051 \| Histone deacetylase inhibition \| 0.2 \| \| 88 \| SIRT1 Inhibitor III \| SIRT1 histone deacetylase inhibition \| 2 \| \| 89 \| Salermide \| SIRT1/2 histone deacetylase inhibition \| 10 \| \| 90 \| SRT1720 \| SIRT1 histone deacetylase activation \| 1 \| \| 91 \| Anacardic acid \| Histone acetyltransferase inhibition \| 5 \| \| 92 \| CTPB \| P300 histone acetyltransferase activation \| 5 \| \| 93 \| JQ1 \| BET bromodomain inhibition \| 0.2 \| \| 94 \| I-BET-762 \| BET bromodomain inhibition \| 0.2 \| \| 95 \| OAC1 \| Epigenetic modulation \| 10 \| \| 96 \| OAC2 \| Epigenetic modulation \| 5 \| \| 97 \| N-oxaloylglycine \| Prolyl 4-hydroxylase inhibition \| 1 \| \| 98 \| Quercetin \| mitochondrial ATPase and phosphodiesterase inhibition \| 1 \| \| 99 \| 2-Deoxy-D-glucose \| Glycolysis inhibition \| 5000 \| \| 100 \| Fasudil (HA-1077) HCl \| ROCK inhibition \| 2 \| \| 101 \| Pyrintegin \| Integrin signaling activation \| 3 \| \| 102 \| Eosin Y Disodium Trihydrate (AMI-5) \| Histone arginine methyltransferase inhibition \| 5 \| \| 103 \| CD1530 \| Potent and selective RARγ agonist \| 0.1 \| \| 104 \| DY131 \| A selective agonist at ERRβ and ERRγ \| 10 \| \| 105 \| DLPC \| NR5A2 agonist \| 50 \| \| 106 \| Ch55 \| RAR-a/b activator \| 1 \| \| 107 \| SMER28 \| regulator of autophagy \| 10 \| \| 108 \| AS8351 \| KDM5B inhibitor \| 1 \| \| 109 \| Resveratrol \| SIRT1 histone deacetylase activation \| 5 \| \| 110 \| Pifithrin-α (PFTα) \| P53 inhibition \| 5 \| \| 111 \| Pifithrin-μ \| P53 inhibition \| 5 \| \| 112 \| 17β-Estradiol \| ESR activator \| 10 \| \| 113 \| Torkinib (PP242) \| mTOR inhibitor \| 1 \| \| 114 \| BMS-189453 (RAi) \| Synthetic retinoid and RARβ agonist; also RARαand RARγ antagonist \| 1 \| \| 115 \| LY294002 \| PI3K inhibitor \| 1 \| \| 116 \| LOE 908 hydrochloride \| a broad spectrum cation channel blocker \| 5 \| \| 117 \| A23187, free acid \| Calcium ionophore \| 1 \| \| 118 \| Phorbol 12-myristate 13-acetate (PMA) \| Protein kinase C activator \| 0.1 \| \| 119 \| SNAP \| A stable analog of endogenous S-nitroso compounds \| 100 \| \| 120 \| SR 202 \| Selective PPARγ antagonist \| 5 \| \| 121 \| LE 135 \| Retinoic acid antagonist \| 5 \| \| 122 \| NKH 477 \| Water-soluble analog of forskolin \| 5 \| \| 123 \| PAC-1 \| Activator of procaspase-3; pro-apoptotic \| 5 \| \| 124 \| GSK 4716 \| Selective agonist of ERRβ and ERRγ \| 5 \| \| 125 \| ML 228 \| HIF pathway activator \| 5 \| \| 126 \| Acetylcysteine (NAC) \| Glutathione (GSH) precursor and cell permeable antioxidant \| 5 \| \| 127 \| Pentamidine isethionate (PTM) \| inhibits constitutive nitric oxide synthase in the brain and acts as a NMDA glutamate receptor antagonist \| 5 \| \| 128 \| DMOG \| α-KG antagonistand HIF prolylhydroxylase inhibitor \| 5 \| \| 129 \| Roscovitine (Seliciclib,CYC202) \| a potent, selective inhibitor of CDK \| 10 \| \| 130 \| Aloisine A RP107 (CAS 496864-16-5) \| An inhibitor of CDK1, CDK2, CDK5, GSK-3 alpha, and JNK \| 0.1 \| \| 131 \| RPI-1 \| RET Receptor Tyrosine Kinase Inhibitor \| 10 \| \| 132 \| GW3965 HCl \| Active non-steroidal agonist for the liver X receptor (LXR) \| 2 \| \| 133 \| T0901317 \| LXR agonist \| 10 \| \| 134 \| 24(S)-Hydroxycholesterol (EPM-1) \| endogenous agonist for LXR \| 1 \| \| 135 \| Pregnenolone-16α-carbonitrile (PCN) \| PXR (pregnane X receptor) activator \| 10 \| \| 136 \| SR 12813 \| PXR agonist \| 2 \| \| 137 \| Cytosporone B \| Naturally occurring NR4A1 agonist \| 1 \| \| 138 \| Ciglitazone \| Selective agonist at PPARγ \| 10 \| \| 139 \| SR 1664 \| High affinity PPARγ ligand; blocks Cdk5-dependent PPARγ phosphorylation \| 1 \| \| 140 \| Genistein \| PPARγligand,estrogen receptor ligand and EGFR inhibitor \| 2 \| \| 141 \| Pirfenidone \| Antifibrotic agent \| 10 \| \| 142 \| Nintedanib (BIBF 1120) \| Inhibits multiple tyrosine kinases \| 1 \| \| 143 \| Rosiglitazone \| Potent and selective PPARγ agonist \| 10 \| \| 144 \| Pioglitazone \| Selective PPARγ agonist \| 1 \| \| 145 \| Imatinib (STI571) \| Inhibitor of tyrosine kinases of the TGFβand PDGF pathways \| 10 \| \| 146 \| SIS3 \| Selective Smad3 inhibitor \| 5 \| \| 147 \| Calpeptin \| calpain inhibitor \| 0.1 \| \| 148 \| CITCO \| Constitutive androstane receptor agonist \| 2 \| \| 149 \| Bumetanide (EPM-3) \| MET \| 5 \| \| 150 \| Estradiol valerate (EPM-2） \| MET \| 5 \| \| 151 \| CAS 313981-82-7 (EPM-11) \| MET \| 5 \| \| 152 \| CAS 912791-92-5 (EPM-13) \| MET \| 5 \| \| 153 \| CAS 890825-02-2 (EPM-15） \| MET \| 5 \| \| 154 \| Lanosterol (EPM-4) \| Cholesterol precursor sterol \| 5 \| \| 155 \| Tamoxifen \| Estrogen receptor partial antagonist \| 10 \| \| 156 \| Oxindole I \| A potent, selective inhibitor of VEGF \| 10 \| \| 157 \| Methacycline HCl \| MET \| 5 \| \| 158 \| AUTEN67 \| MTMR inhibitor \| 10 \| \| 159 \| SF51 \| calcium channel atagonist \| 20 \| \| 160 \| NC043 \| USP30 inhibitor \| 2 \| \| 161 \| EPI743 \| CoQ10 analogue \| 1 \| \| 162 \| Urolithin A \| Mitophagy inducer \| 50 \| \| 163 \| Doxycycline hyclate \| an inhibitor of matrix metallo-proteinases (MMP) \| 1 \| \| 164 \| LiCL \| inhibits the replication of type 1 and type 2 Herpes \| 2 \| \| 165 \| Nicotinamide \| PARP-1 inhibitor \| 10 \| \| 166 \| sphingosine-1-phosphate \| A lipid second messenger that binds to S1P1 and S1P3 receptors \| 0.5 \| \| 167 \| Ponasterone A \| derivates of ecdysone, a kind of insect hormone \| 1 \| \| 168 \| Blebbistain \| myosin II ATPase inhibitor \| 2 \| \| 169 \| RO4929097 \| γ secretase inhibitor, Notch inhibitor \| 10 \| \| 170 \| Oleoyl-L-a-lysophosphatidic acidic sodium salt \| a proliferative and anti-apoptotic factor, signaling for Pl3K-mediated regulation of cell activity. \| 5 \| \| 171 \| D-Fructose 1,6-bisphosphate trisodium salt \| An allosteric activator of enzymes \| 5 \| \| 172 \| EPZ015666 \| Prmt5 inhibitor \| 5 \| \| 173 \| MI-2 \| MLL2(MLL) inhibitor \| 10 \| \| 174 \| SGI-1027 \| Dnmt3A/B inhibitor \| 100 \| \| 175 \| MK-5108 (VX-689) \| AURORA_A inhibitor \| 1 \| \| 176 \| AZD1152-HQPA \| AURORA_B inhibitor \| 1 \| \| 177 \| 1400W dihydrochloride \| iNOS inhibitor \| 100 \| \| 178 \| FH1(BRD-K4477) \| hepatocyte maturation activator \| 25 \| \| 179 \| FPH1 (BRD-6125) \| hepatocyte maturation activator \| 25 \| \| 180 \| QNZ \| NF-kB inhibitor \| 5 \| \| 181 \| Wortmannin \| PI3K inhibitor \| 1 \| \| 182 \| Dexamethasone \| Dexamethasone \| 0.1 \| \| 183 \| Luteolin \| TNF-α, IL-6, NF-Κb, AP-1 inhibitor \| 7.5 \| \| 184 \| WL5A5 \| MST inhibitor \| 1 \| \| 185 \| Clomiphene citrate \| estrogen agonist \| 2 \| \| 186 \| Niclosamide \| anthelmintic and potential antineoplastic activity \| 0.1 \| \| 187 \| Kinetin \| a geroprotector and a cytokinin \| 20 \| \| 188 \| Fluphenazine \| a phenothiazine and antipsychotic agent \| 10 \| \| 189 \| PFI 3 \| an azabicycloalkane \| 2 \| \| 190 \| LY2090314 \| GSK-3α/β inhibitor \| 5 \| \| 191 \| CP2 \| KDM4 inhibitor \| 5 \| \| 192 \| Dorsomorphin 2HCl (Compound C) \| a potent and selective inhibitor of AMPK \| 2 \| \| 193 \| XAV939 \| Tankyrase1/2 inhibitor \| 5 \| \| 194 \| IWP2 \| Porcn mediated Wnt palmitoylation \| 5 \| \| 195 \| A-485 \| p300/CBP selective catalytic inhibior \| 10 \| \| 196 \| ascorbic acid \| increases the active iron (Fe2+) required for the TET \| 10 \| \| 197 \| CHIR-98014 \| GSK-3α/β inhibitor \| 10 \| \| 198 \| adenosine \| metabolite \| 1 \| \| 199 \| 4-Hydroxyquinoline \| metabolite \| 100 \| \| 200 \| Fumaric acid \| metabolite \| 100 \| \| 201 \| SAG \| Smo receptor agonist \| 1 \| \| 202 \| TTFA \| complex II inhibitior \| 5 \| \| 203 \| ISRIB \| inhibition of ISR \| 5nM \| \| 204 \| Eriodictyol 7-O-glucoside \| unknown \| 10 \| \| 205 \| vitamin K1 \| photosynthesis \| 10 \| \| 206 \| WP1066 \| apoptosis \| 5 \| \| 207 \| ciclopirox \| iron chelator \| 2 \| \| 208 \| BIRB \| P38 \| 10 \| \| 209 \| Bergapten \| cell replication \| 5 \| \| 210 \| BIA 2-093 \| antiepileptic \| 5 \| \| 211 \| Ketanserin tartrate \| 5-HT2A \| 5 \| \| 212 \| Doxazosin Mesylate \| apoptosis \| 5 \| \| 213 \| Elesclomol (STA-4783) \| oxidative stress inducer \| 5 \| \| 214 \| Flupirtine maleate \| analgesic \| 1ug/ml \| \| 215 \| Ropinirole HCl \| anti-oxidant \| 5 \| \| 216 \| T0070907 \| PPARγ \| 20 \| \| 217 \| Loteprednol etabonate \| antiinflammation \| 5 \| \| 218 \| Epoxomicin \| proteasome inhibitor \| 5 \| \| 219 \| Oleuropein \| proteasome activator \| 5 \| \| 220 \| MG132 \| proteasome inhibitor \| 5 \| \| 221 \| Crizotinib \| ALK inhibitor \| 5 \| \| 222 \| Ceritinib \| ALK inhibitor \| 5 \| \| 223 \| Brigatinib \| ALK inhibitor \| 5 \| \| 224 \| Lapatinib \| RTK inhibitor \| 5 \| \| 225 \| Vemurafenib \| MAPK inhibitor \| 10 \| \| 226 \| ABT-263 \| anti-apoptotic protein inhibitor \| 5 \| \| 227 \| WM-1119 \| KAT6A inhibitor \| 1 \| \| 228 \| Dabrafenib \| MAPK inhibitor \| 1 \| \| 229 \| Trametinib \| MAPK inhibitor \| 1 \| \| 230 \| Timapiprant \| DP2 antagonist \| 5 \| \| 231 \| Ridaforolimus (deforolimus) \| mTOR inhibitor \| 5 \| \| 232 \| GW441756 \| Tropomyosin-related kinase A (TrkA) inhibitor \| 5 \| \| 233 \| ZLN005 \| PGC-1α transcriptional activator \| 10 \| \| 234 \| Tracheloside \| decrease the activity of alkaline phosphatase \| 10 \| \| 235 \| Periplocin \| activation of Src/ERK,PI3K/Akt \| 10 \| |
| --- | --- | --- | --- | --- | --- | --- | --- | --- | --- | --- | --- | --- | --- | --- | --- | --- | --- | --- | --- | --- | --- | --- | --- | --- | --- | --- | --- | --- | --- | --- | --- | --- | --- | --- | --- | --- | --- | --- | --- | --- | --- | --- | --- | --- | --- | --- | --- | --- | --- | --- | --- | --- | --- | --- | --- | --- | --- | --- | --- | --- | --- | --- | --- | --- | --- | --- | --- | --- | --- | --- | --- | --- | --- | --- | --- | --- | --- | --- | --- | --- | --- | --- | --- | --- | --- | --- | --- | --- | --- | --- | --- | --- | --- | --- | --- | --- | --- | --- | --- | --- | --- | --- | --- | --- | --- | --- | --- | --- | --- | --- | --- | --- | --- | --- | --- | --- | --- | --- | --- | --- | --- | --- | --- | --- | --- | --- | --- | --- | --- | --- | --- | --- | --- | --- | --- | --- | --- | --- | --- | --- | --- | --- | --- | --- | --- | --- | --- | --- | --- | --- | --- | --- | --- | --- | --- | --- | --- | --- | --- | --- | --- | --- | --- | --- | --- | --- | --- | --- | --- | --- | --- | --- | --- | --- | --- | --- | --- | --- | --- | --- | --- | --- | --- | --- | --- | --- | --- | --- | --- | --- | --- | --- | --- | --- | --- | --- | --- | --- | --- | --- | --- | --- | --- | --- | --- | --- | --- | --- | --- | --- | --- | --- | --- | --- | --- | --- | --- | --- | --- | --- | --- | --- | --- | --- | --- | --- | --- | --- | --- | --- | --- | --- | --- | --- | --- | --- | --- | --- | --- | --- | --- | --- | --- | --- | --- | --- | --- | --- | --- | --- | --- | --- | --- | --- | --- | --- | --- | --- | --- | --- | --- | --- | --- | --- | --- | --- | --- | --- | --- | --- | --- | --- | --- | --- | --- | --- | --- | --- | --- | --- | --- | --- | --- | --- | --- | --- | --- | --- | --- | --- | --- | --- | --- | --- | --- | --- | --- | --- | --- | --- | --- | --- | --- | --- | --- | --- | --- | --- | --- | --- | --- | --- | --- | --- | --- | --- | --- | --- | --- | --- | --- | --- | --- | --- | --- | --- | --- | --- | --- | --- | --- | --- | --- | --- | --- | --- | --- | --- | --- | --- | --- | --- | --- | --- | --- | --- | --- | --- | --- | --- | --- | --- | --- | --- | --- | --- | --- | --- | --- | --- | --- | --- | --- | --- | --- | --- | --- | --- | --- | --- | --- | --- | --- | --- | --- | --- | --- | --- | --- | --- | --- | --- | --- | --- | --- | --- | --- | --- | --- | --- | --- | --- | --- | --- | --- | --- | --- | --- | --- | --- | --- | --- | --- | --- | --- | --- | --- | --- | --- | --- | --- | --- | --- | --- | --- | --- | --- | --- | --- | --- | --- | --- | --- | --- | --- | --- | --- | --- | --- | --- | --- | --- | --- | --- | --- | --- | --- | --- | --- | --- | --- | --- | --- | --- | --- | --- | --- | --- | --- | --- | --- | --- | --- | --- | --- | --- | --- | --- | --- | --- | --- | --- | --- | --- | --- | --- | --- | --- | --- | --- | --- | --- | --- | --- | --- | --- | --- | --- | --- | --- | --- | --- | --- | --- | --- | --- | --- | --- | --- | --- | --- | --- | --- | --- | --- | --- | --- | --- | --- | --- | --- | --- | --- | --- | --- | --- | --- | --- | --- | --- | --- | --- | --- | --- | --- | --- | --- | --- | --- | --- | --- | --- | --- | --- | --- | --- | --- | --- | --- | --- | --- | --- | --- | --- | --- | --- | --- | --- | --- | --- | --- | --- | --- | --- | --- | --- | --- | --- | --- | --- | --- | --- | --- | --- | --- | --- | --- | --- | --- | --- | --- | --- | --- | --- | --- | --- | --- | --- | --- | --- | --- | --- | --- | --- | --- | --- | --- | --- | --- | --- | --- | --- | --- | --- | --- | --- | --- | --- | --- | --- | --- | --- | --- | --- | --- | --- | --- | --- | --- | --- | --- | --- | --- | --- | --- | --- | --- | --- | --- | --- | --- | --- | --- | --- | --- | --- | --- | --- | --- | --- | --- | --- | --- | --- | --- | --- | --- | --- | --- | --- | --- | --- | --- | --- | --- | --- | --- | --- | --- | --- | --- | --- | --- | --- | --- | --- | --- | --- | --- | --- | --- | --- | --- | --- | --- | --- | --- | --- | --- | --- | --- | --- | --- | --- | --- | --- | --- | --- | --- | --- | --- | --- | --- | --- | --- | --- | --- | --- | --- | --- | --- | --- | --- | --- | --- | --- | --- | --- | --- | --- | --- | --- | --- | --- | --- | --- | --- | --- | --- | --- | --- | --- | --- | --- | --- | --- | --- | --- | --- | --- | --- | --- | --- | --- | --- | --- | --- | --- | --- | --- | --- | --- | --- | --- | --- | --- | --- | --- | --- | --- | --- | --- | --- | --- | --- | --- | --- | --- | --- | --- | --- | --- | --- | --- | --- | --- | --- | --- | --- | --- | --- | --- | --- | --- | --- | --- | --- | --- | --- | --- | --- | --- | --- | --- | --- | --- | --- | --- | --- | --- | --- | --- | --- | --- | --- | --- | --- | --- | --- | --- | --- | --- | --- | --- | --- | --- | --- | --- | --- | --- | --- | --- | --- | --- | --- | --- | --- | --- | --- | --- | --- | --- | --- | --- | --- | --- | --- | --- | --- | --- | --- | --- | --- | --- | --- | --- | --- | --- | --- | --- | --- | --- | --- | --- | --- | --- | --- | --- | --- | --- | --- | --- | --- | --- | --- | --- | --- | --- | --- | --- | --- | --- | --- | --- | --- | --- | --- | --- | --- | --- | --- | --- | --- | --- | --- | --- | --- | --- | --- | --- | --- | --- | --- | --- | --- | --- | --- | --- | --- | --- | --- | --- | --- | --- | --- | --- | --- | --- | --- | --- | --- | --- | --- | --- | --- | --- | --- | --- | --- | --- | --- | --- | --- | --- | --- | --- | --- | --- | --- | --- | --- | --- | --- | --- | --- | --- | --- | --- | --- | --- | --- | --- | --- | --- | --- | --- | --- | --- | --- | --- | --- | --- | --- | --- | --- | --- | --- | --- | --- | --- | --- | --- | --- | --- | --- | --- | --- | --- | --- | --- | --- | --- | --- | --- |
